# Reversing the Psychiatric Effects of Neurodevelopmental Cannabinoid Exposure: Exploring Pharmacotherapeutic Interventions for Symptom Improvement

**DOI:** 10.3390/ijms22157861

**Published:** 2021-07-23

**Authors:** Marta De Felice, Steven R. Laviolette

**Affiliations:** 1 Addiction Research Group, Department of Anatomy and Cell Biology, Schulich School of Medicine & Dentistry, University of Western Ontario, London, ON N6A 5C1, Canada; mdefelic@uwo.ca; 2 Department of Psychiatry, Schulich School of Medicine & Dentistry, University of Western Ontario, London, ON N6A 5C1, Canada

**Keywords:** cannabis, marijuana, THC, endocannabinoid system, adolescence, neurodevelopment, psychiatric disorders, mesocorticolimbic system

## Abstract

Neurodevelopmental exposure to psychoactive compounds in cannabis, specifically THC, is associated with a variety of long-term psychopathological outcomes. This increased risk includes a higher prevalence of schizophrenia, mood and anxiety disorders, and cognitive impairments. Clinical and pre-clinical research continues to identify a wide array of underlying neuropathophysiological sequelae and mechanisms that may underlie THC-related psychiatric risk vulnerability, particularly following adolescent cannabis exposure. A common theme among these studies is the ability of developmental THC exposure to induce long-term adaptations in the mesocorticolimbic system which resemble pathological endophenotypes associated with these disorders. This narrative review will summarize recent clinical and pre-clinical evidence that has elucidated these THC-induced developmental risk factors and examine how specific pharmacotherapeutic interventions may serve to reverse or perhaps prevent these cannabis-related risk outcomes.

## 1. Introduction

Cannabis is one of the most widely used psychoactive drugs for recreational and medical purposes. The increasing legalization of marijuana around the world strengthens the need to pursue clinical and pre-clinical investigations into its potential detrimental consequences, especially in developing brains. Adolescence is a period of profound neurodevelopmental vulnerability in which crucial structural and functional neural changes occur, but it is also a common life-stage for drug experimentation. Analysis of cannabis use revealed that in European countries up to one-third of adolescents (12 to 17 years) smoke cannabis and 1.2% of them consume it regularly [1], whereas a progressive increase of use from 14% to 44% was reported among high school American teens [2]. In addition, a recent Canadian Cannabis Survey reported that, in 2020, the percentage of cannabis use in youth (16 to 24 years) was approximately two-fold higher than in older people (>25 years), and 31% of people aged 16 to 19 and 20 to 24 reported increased consumption of cannabis due to COVID-19 [3]. Such findings were concomitant with a reduction in perceived risks, since fewer participants (66% in 2020 vs. 75% in 2019) believed that daily cannabis smoke can increase the vulnerability for mental health disorders [3]. These trends are highly concerning given that age of onset, frequency, and duration of exposure have been reported as significant risk factors for a wide array of cannabis-related pathophenotypes. In fact, sustained exposure to the main psychoactive marijuana phytochemical, Δ-9-tetrahydrocannabinol (THC), induces overstimulation of cannabinoid receptors type 1 (CB1Rs) and may profoundly impact the physiological development of the endocannabinoid system (ECS). Notably, while CB1R endogenous ligands, such as anandamide (AEA), are subjected to metabolic control through a complex system of biosynthetic and degradative enzymes, THC largely escapes these neuroregulatory control mechanisms, resulting in more sustained and less regulated neurophysiological effects [4].

Clinical and pre-clinical evidence has revealed numerous long-lasting detrimental effects induced by chronic cannabis use during adolescence. Specifically, developmental THC exposure has been associated with a wide range of cognitive impairments and enhanced vulnerability for neuropsychiatric disorders, concomitant with significant neuroanatomical abnormalities and maladaptations in the mesocorticolimbic system [5,6,7].

Importantly, the relative potency of THC appears to have a crucial role in cannabis pathology. Over the past decades, THC concentration has increased progressively while no changes were observed in the levels of cannabidiol (CBD) [8,9], the main non-psychoactive constituent of cannabis and which has been found to protect against various THC-related effects [10,11,12]. Young subjects consuming high-THC cannabis strains (e.g., *sinsemilla*) were more likely to develop anxiety and cannabis use disorder [13] as well as to experience psychiatric-like symptoms [14,15] and frequent relapses [16]. 

Therefore, adolescence represents a vulnerable developmental window during which external insults can induce profound remodeling in brain maturation and functionality, leading to pathological phenotypes later in life. In this narrative review, we provide an overview of long-lasting behavioral alterations and brain development aberrations following chronic cannabinoids exposure during adolescence in human and animal models. Moreover, we identify pharmacotherapeutic targets aiming to minimize, reverse, or prevent these cannabis-related pathological outcomes.

## 2. Long-Lasting Consequences of Sustained Cannabis Use during Adolescence: Clinical Evidence

Over the last decades, a plethora of epidemiologic studies have shown an association between sustained exposure to cannabis during adolescence and an enhanced vulnerability for various neuropsychiatric disorders later in life. This effect was reported for the first time in a large-scale population analysis in 1987 by Andréasson and colleagues, which demonstrated that heavy consumers of cannabis exhibited six-fold higher risks than non-users to develop schizophrenia at adulthood [17]. The age of onset of cannabis use has been considered a significant risk factor for psychopathological outcomes. Indeed, both positive and negative dimensions of psychosis were observed in subjects using cannabis for the first time before the age of 16 [18]. In addition, early cannabis consumption has been associated with greater risk of depression phenotypes and anxiety [19,20] as well as cognitive alterations [21]. Chronic cannabis exposure before age 17 has been found to induce long-lasting neurocognitive decrements in IQ, working memory, executive functioning, decision making, impulsivity, attention, and scholastic performances [22,23,24]. A correlation between cognitive impairments and neurostructural maladaptations has been suggested by Jacobus and colleagues in prospective investigations over 3 and 6 years. These authors reported that cannabis users exhibited not only poorer attention and memory, but also reduced white and gray matter integrity as well as thicker frontal and temporal cortices [25]. Consistently, white matter integrity was reduced in prefrontal, limbic, parietal, and cerebellar tracts in young marijuana users [26], and an opposite pattern in cortical thickness was associated with the onset of use, highlighting thicker cortices in early- vs. late-onset users [27]. Magnetic resonance imaging scans also revealed that heavy marijuana consumption induced increased thickness in the bilateral lingual, right superior temporal, right inferior parietal, and left paracentral regions, and a decreased in right caudal middle frontal, bilateral insula, and bilateral superior frontal cortices [28].

Other studies have also provided evidence of how marijuana exposure may impact brain structure development during neurodevelopmental epochs. For example, analyses of brain volume measures revealed smaller cortical gray matter and larger white matter volumes in marijuana users who started before age 17 [29]. Moreover, morphological changes have been detected in thalamus, hippocampus, parahippocampus, and amygdala [30,31,32,33], and profound impairments in axonal connectivity were observed in the corpus callosum and in the right fimbria of the hippocampus [34], which may underlie the long-term cognitive impairments observed in cannabis-related pathology.

At the neurochemical level, dysfunctions in the balance between the excitatory and inhibitory pathways have been considered as potential mechanisms underlying cannabis-related disturbances. Indeed, repeated stimulation of CB1Rs during brain maturation may interfere with normal neurodevelopmental processes, especially in frontal cortex, hippocampus and striatum, in which CB1Rs expression progressively increases until adulthood [35]. Although no clinical studies to our knowledge have yet reported long-lasting consequences on GABAergic and glutamatergic systems following adolescent cannabis exposure, aberrations have been observed in young chronic smokers [36] and in cannabis users in an early stage of psychosis [37]. Such abnormalities are consistent with those observed in neuropsychiatric disorders. Indeed, post-mortem analyses in schizophrenic patients revealed a reduction in cortical levels of the enzyme glutamate decarboxylase (GAD-67), which mediate GABA synthesis and release [38]. Moreover, decreased in the levels of GAT-1 and GABA_A_ receptor subunits have been reported [39,40,41]. Similar alterations in GAD-67 and GABA_A_ receptors were also observed in patients with major depression [42,43,44].

Furthermore, the consequences of adolescent marijuana use on dopaminergic system function have been investigated in both clinical and pre-clinical studies. For example, sustained cannabis exposure has been associated with reduced dopamine (DA) release within the striatum, which was related to the onset and duration of use [45,46]. Moreover, the Catechol-Methyl-Transferase (COMT) and Akt genes have been examined, due to their role in DA regulation and psychosis. Caspi and colleagues demonstrated that a functional polymorphism in COMT gene moderates the emergence of psychosis at adulthood, following chronic adolescent cannabis use [47]. This phenomenon was not observed after acute consumption in young smokers, which was mediated instead by a genetic variation of the Akt gene [48]. Consistently, the same polymorphism of the Akt gene has been directly linked to enhanced susceptibility for cannabis-related psychopathology [49]. In addition, investigations of brain-derived neurotrophic factor (BDNF) gene, a neurotrophin involved in neuronal growth, differentiation, and survival, pointed out a deregulation in BDNF production as a consequence of frequent cannabis use in adolescence [50]. Cannabis use at young age and BDNF variations have been also correlated with the onset of psychosis [51].

Taken together, this evidence highlights the role of neuronal and genetic markers in the association between early cannabis use and vulnerability to neuropsychiatric disorders, providing potential approaches for novel therapeutic strategies to prevent and/or reverse the pathophysiological sequelae induced by chronic marijuana consumption during adolescence. However, the majority of human clinical studies are correlational in nature and are limited in their ability to dissociate causal vs. correlative measures, either temporally or functionally. Accordingly, pre-clinical studies primarily using rodent models of adolescent brain development have been exceptionally important for identifying the specific functional roles in various biomarkers linked to adolescent cannabinoid exposure. In addition, there is now emerging coherence between many such pre-clinical studies and reported effects in clinical populations, leading to increased opportunities for the identification of pharmacological or other therapeutic interventions aimed at reversing the pathophysiological effects of adolescent cannabinoid exposure.

## 3. Effects of THC Exposure during Adolescence: Insight from Neurodevelopmental Animal Models 

### 3.1. Risks for Schizophrenia and Cognitive Impairments 

Pre-clinical investigations on adolescent neurodevelopmental cannabis exposure have been crucial to model cannabinoid-related endophenotypes comparable to those reported in schizophrenia and other neuropsychiatric diseases. Studies in animal models provide the advantage of identifying cannabinoid-induced neuroadaptations, while precisely controlling drug concentration and the age window of exposure, as well as allowing for mechanistic approaches for preventing or reverting observed psychopathological phenotypes. The need for improving the translational value of these models have led to the development of more precise definitions of adolescence in rodents as the periods between postnatal days (PND) 21 and 60, which can be further dissected in three phases: early, middle, and late adolescence, starting at PND 21, PND 34, and PND 46, respectively [52].

While schizophrenia is undoubtedly a uniquely human disease, various behavioral paradigms with high validity have been used to investigate schizophrenia-like features following adolescent cannabinoid exposure. For example, the measurement of sensorimotor gating in prepulse inhibition (PPI) paradigms is one of the most widely used assays to investigate sensory filtering impairments in various neuropsychiatric disorders, including schizophrenia and autism. Indeed, the inability to filter out irrelevant sensory information is a core endophenotype in schizophrenia [53]. Chronic THC exposure during middle adolescence in rodents (PND 35–45) has been found to significantly disrupt sensorimotor gating at adulthood [54,55]. Notably, this effect was neither observed directly after adolescent THC exposure [55], nor when THC was administered to adult rats (PND 65–75) [54]. 

Social cognition and memory impairments are other core endophenotypes of schizophrenia disorders [56]. Neurodevelopmental THC exposure during adolescence in animal models has been found to induce cognitive schizophrenia-like deficits [57,58]. For example, significant long-term abnormalities in social interaction memory were observed in adolescent rats chronically treated with THC [54,59]. Similarly, short-term and working memory performances were altered when specific tasks (e.g., novel and spatial object recognition, radial arm maze, T maze, Morris water maze) were carried out in both males and females [59,60,61,62,63]. These THC-induced effects on memory have not been reported consistently among studies [64,65,66,67]. However, the route of administration and dosing regimen of THC used across study protocols, as well as variations in behavioral paradigm techniques may account for such discrepancies. Relevant long-lasting deficits have been also observed during the paired-associates learning task, which assess for associative learning and visual memory deficits. Specifically, adolescent THC-exposed rats required more trials than controls to reach the accuracy criterion of 80% at adulthood [55]. 

### 3.2. Risks for Depressive Like-Phenotype and Long-Term Anxiety

Considerable evidence has shown that adolescent cannabinoid exposure in rodents leads to long-term dysregulation in emotional processing, resulting in anxiety and depressive-like symptoms. In fact, adult rats chronically exposed to THC during adolescence, when placed in an inescapable water cylinder during the Porsolt Forced Swim Test (FST), stopped moving earlier and/or spent longer time immobile [57,58,68]. These behaviors, which are considered as measures of despair and resignation, have been found to be greater in females [69,70]. Furthermore, adolescent THC exposure has been found to induce anhedonia-like behaviors, demonstrated by reduced interest in rewarding stimuli measured in sucrose and palatable food preference tests [68,70,71]. These findings support the role of cannabis consumption during adolescence in the susceptibility for developing mood disorders at adulthood, also showing consistency with the remarkable gender differences reported in depression.

In regard to anxiety-related disorders, results reported following adolescent THC exposure are less consistent. Chronic THC treatment during adolescence induced long-lasting anxiety in the light-dark box test [54], a paradigm based on the aversion of rodents to bright environments. Nonetheless, when other assays were performed, adolescent THC has been found to induce either no changes, anxiety- or anxiolytic-like effects. Specifically, adult rats treated with low doses of THC from PND 30 to 50 spent less time and made less entries in the open arms during the Elevated Plus Maze test [68]. On the other hand, studies reported no long-term differences in THC-exposed males and females [71,72] as well as in C57Bl/6J and DBA/2J male mice [63,65]. In addition, THC has been also found to induce strain-specific anxiolytic effects in Lewis rats, which spent more time in the open arms compared both their vehicle counterpart and THC-exposed Fischer344 rats [73]. These findings may highlight the role of genetic backgrounds and differential vulnerabilities in THC-induced maladaptations. Indeed, one study reported no affective dysregulations in adult Long-Evans rats following chronic adolescent THC exposure [74]. Lastly, contrasting findings have been reported when thigmotaxis in the Open Field test was considered. This anxiety index is based on the preference of rodents to stay close to the walls rather than exploring the open arena of an enclosed test environment, related to a rodent’s natural avoidance of predator vulnerability. Using this assay, THC exposure during adolescence has variably been reported to reduce [75], increase [60], or not alter [68,71] the thigmotaxis index at adulthood, again suggesting a complex interplay between genetic background and relative vulnerability to THC-induced anxiety phenotypes.

## 4. Neurobiological Mechanisms Underlying THC-Related Detrimental Consequences

**Figure 1:**
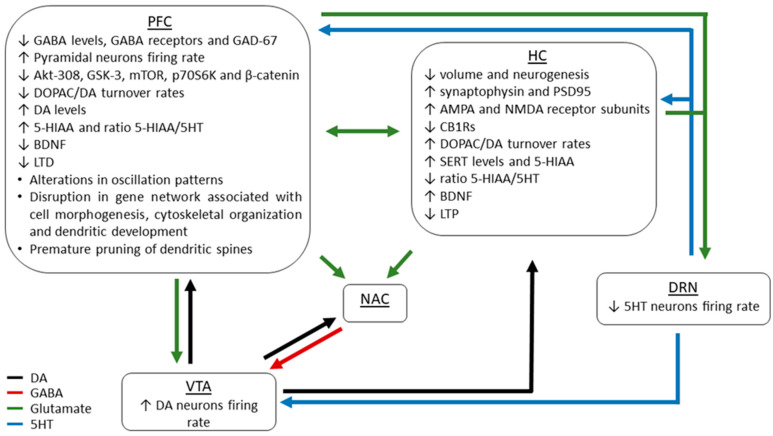
Schematic circuit diagram illustrating the long-lasting alterations in brain regions affected by adolescent THC exposure.

Several neural and molecular pathways have been investigated to elucidate the mechanisms underlying THC-induced pathology (Figure 1). Chronic cannabinoid exposure is able to downregulate CB1R expression in several brain regions [76,77,78], interfering with the normal development of the endocannabinoid system and neurocircuitries during adolescence. This impact on neural remodeling may lead to the emergence of neuropsychiatric phenotypes and negatively affect long-term cognitive processing, mainly mediated by the prefrontal cortex (PFC). Indeed, THC exposure during adolescence has been found to alter the PFC pyramidal neurons, disrupting the gene networks associated with cell morphogenesis, cytoskeletal organization and dendritic development as well as provoking a premature pruning of dendritic spines [79]. Moreover, aberrations in cortical glutamatergic and GABAergic systems have been previously observed in rodent models of adolescent THC exposure. For example, several pre-clinical studies have demonstrated that THC exposure during adolescence induced a decrease of basal GABA levels and GABA receptor proteins as well as a remarkable loss of GAD-67 [76,80,81], associated with hyperactive states of PFC pyramidal neurons [59]. These disturbances in the inhibitory/excitatory balance have been correlated with abnormal oscillation patterns [81], which are directly implicated in sensory processing and cognitive impairments [82,83]. Moreover, the increased firing rate of PFC glutamatergic neurons following adolescent THC exposure has been found to drive a sub-cortical DAergic hyperactivity in ventral tegmental area (VTA), underlying schizophrenia-like phenotypes [54]. In support of this, previous investigations have shown that activation of CB1Rs in PFC modulated emotional and memory salience through functional interaction with the mesolimbic DAergic system [84]. Such disturbances in DAergic signaling may result from neurodevelopmental alterations in DA downstream molecular pathways. In particular, adolescent THC exposure has been shown to significantly down-regulate the PFC expression of several molecular substrates related to psychiatric disorders, such as Akt-308, GSK-3, mTOR, p70S6K, and β-catenin [54]. 

Furthermore, THC-induced deficits in cognitive and memory functions could be attributed to disruptions in the hippocampus (HC) and PFC connection pathways. For example, sustained adolescent THC exposure has been found to lead to sex-dependent downregulation and disrupted functionality of CB1Rs [85] as well as reduction in hippocampal volume and neurogenesis [62,70]. In addition, Zamberletti and colleagues reported an enhanced expression of pre- and post-synaptic hippocampal biomarkers (e.g., synaptophysin and PSD95) and specific AMPA and NMDA receptor subunits, concomitant with neuroinflammatory processes [86]. At the functional level, long-term potentiation (LTP) and long-term depression (LTD), two processes critical for memory-related synaptic plasticity, have been reported to be impaired by adolescent THC exposure in dental gyrus (DG) and PFC, respectively [87]. Moreover, THC exposure has been shown to induce abnormal changes in DA transmission as well as in serotonin (5HT) transporter (SERT) expression levels and serotonergic activity in both the PFC and HC. Specifically, in male rats, DOPAC/DA turnover rates were increased in HC and decreased in PFC, while tissue DA levels were increased only in PFC [62]. In addition, SERT levels were enhanced only in HC, the 5HT metabolite 5-HIAA was significantly increased in both PFC and HC, while the ratio 5-HIAA/5HT was increased in PFC but decreased in HC [60]. These findings were concomitant with changes in BDNF protein expression levels PFC and HC [62], revealing sex-specific differences exclusively in PFC [60]. 

Due to the relevance of these neural pathways in cognitive processes, such changes in the neurobiology of PFC and HC may represent specific biomarkers for THC-induced memory impairments. In addition, dysfunction in HC may also account for the affective and emotional impairments induced by adolescent THC exposure, due to its ability to regulate mesolimbic DA activity states. Indeed, the pathway ventral HC-nucleus accumbens (NA) has been previously investigated for its pro-depressant role in depressive-like animal model [88]. Although no study to our knowledge has yet studied the HC-NA circuit specifically following THC exposure, it has been shown that acute intra-HC cannabinoid receptor activation can potently modulate neural activity states within the NA shell (NAsh) and controls the formation of emotional associative memory [89]. In addition, local administration of THC in ventral HC was found to induce anxiety-related phenotypes, associated with increased VTA DA activity, decreased VTA GABAergic, and disruption in β, γ, ε oscillation patterns, through an ERK-signaling mechanism [90]. Notably, abnormalities in synaptic excitatory/inhibitory balance in HC and PFC as well as the hyper-DAergic activity in VTA induced by THC may in turn impact on dorsal raphe nucleus (DRN) activity. Specifically, a recent study has demonstrated that chronic THC exposure during adolescence induced persistent anxious and depressive-like behaviors, concomitant with a hypoactivity of 5HT neurons in DRN [68]. 

Taken together, the evidence highlighted above underscores that the cognitive impairments, anxiety, as well as psychiatric-like manifestations associated with neurodevelopmental THC exposure may result from profound maladaptations in a variety of neural circuits and associated molecular signaling pathways, all of which concomitantly serve as important biomarkers for various neuropsychiatric disorders. 

## 5. Therapeutic Perspective: Potential Intervention for Mitigate the Neuropsychiatric Risks

According to the literature reviewed above, sustained cannabis exposure during adolescence induces long-lasting cognitive impairments and neuropsychiatric-like phenotypes, associated with robust dysfunction of the mesocorticolimbic system. Functionally, the profound dysregulation in sub-cortical DAergic signaling has been associated with disturbances in GABAergic vs. glutamatergic balance in the PFC, leading to dysregulated control of VTA DA transmission via PFC > VTA pathways [81,84]. That considered, novel pharmacotherapeutic interventions targeting the cortical inhibitory/excitatory signaling have now been examined aimed to either minimize, revert, or prevent such cannabis-induced detrimental effects. Renard and colleagues [59] demonstrated that THC-induced neuronal and behavioral abnormalities could be reversed in adulthood by pharmacologically restoring physiological GABA tone directly in the PFC. Specifically, pharmacological agonist activation of GABA_A_ receptors in the PFC at adulthood reversed short-term and social memory impairments as well as reduced anxiety and alterations in exploratory behavior induced by THC exposure during adolescence. Notably, these manipulations of PFC GABAergic neurotransmission also normalized the long-lasting sub-cortical DAergic hyperactivity (Table 1). These findings corroborate the crucial role of cortical GABAergic hypofunction in adolescent THC-induced pathological outcomes and underscore the potential therapeutic benefits of targeting cortical GABAergic tone to mitigate cannabis-related neuropsychiatric side-effects [81]. Importantly, these promising benefits of GABAergic compounds have been already observed in schizophrenic patients, in which the administration of a benzodiazepine-like agent selective for GABA_A_ receptors was shown to improve cognitive performance and attenuated schizophrenia-related abnormalities in frontal gamma oscillatory activity [91]. Future studies are required to see if similar interventions into the GABAergic system may be therapeutic for the reversal of cannabis-induced neuropsychiatric side-effects following long-term adolescent exposure.

Concomitant to the loss of GABAergic inhibition, chronic THC exposure during adolescence was found to induce a remarkable potentiation in firing and bursting activity of PFC pyramidal neurons [54,59], consistent with the dysregulation of GABA/Glutamate balance within this region. Thus, modulation of glutamatergic dysfunction has been proposed as a potential pharmacological intervention to prevent or reverse THC-induced psychopathology. Specifically, L-theanine, an amino acid analogue of L-glutamate and L-glutamine derived from green tea leaves, has been investigated due to its established therapeutic properties in anxiety, schizophrenia and depressive phenotypes [92,93,94,95]. Research in our laboratory has recently demonstrated that co-administration of L-theanine with THC in a neurodevelopmental rodent model of adolescent THC exposure, was able to powerfully block a wide range of THC-induced behavioral abnormalities into adulthood, including anhedonia, anxiety and impairments in memory and sensorimotor gating [96]. These preventative effects of L-theanine might result from its ability to normalize DAergic hyperactivity in the VTA, which in turn could ameliorate the cortical hyper-bursting state, restoring the inhibitory/excitatory balance and the oscillation patterns within PFC. In support of this, we found that L-theanine was able to fully prevent the overactivation of both sub-cortical VTA DAergic neuronal activity and cortical PFC activity states and gamma-oscillatory dysregulation induced by adolescent THC exposure. Furthermore, we found that down-regulation of cortical GSK-3α/β and Akt-Thr308, two critical molecular biomarkers for THC-related neuropsychiatric side effects, was similarly reversed by L-theanine [96] (Table 1). Such decreases in GSK-3α/β and Akt-Thr308 expression levels have been previously associated with hyper-DAergic states, as demonstrated following sustained activation of DA D_2_ receptors [97]. In addition, an in vitro study has demonstrated that L-theanine protects against excess DA-related neurotoxicity through modulation of glutathione levels in astrocytes [98]. This suggests a potential role of astrocytic-glutathione mechanisms underlying mesocorticolimbic PFC/VTA dysregulation induced by hyperactive DAergic drive following adolescent THC exposure. 

Beyond the role of DA, adolescent THC exposure has been found to induce long-term dysregulation in serotonin transmission [60,68], highlighting this system as a potential target for novel therapeutic approaches. In particular, Berthoux and colleagues [99] have shown that co-administration during adolescence of THC and the 5HT_6_ receptor antagonist SB258585, was able to prevent long-lasting cognitive impairments and aberrations in cortical networks, such as sustained activation of mTOR, excitatory/inhibitory transmission imbalances as well as changes in the intrinsic properties of cortical pyramidal neurons and LTD (Table 1). These THC-induced abnormalities were abolished also following concomitant treatment with the mTOR inhibitor rapamycin and were not detected in 5HT_6_ receptor deficiency mice, supporting the crucial role of these receptors in THC-related pathology. Consistently, antagonism of 5HT_6_ receptor has been previously observed to be effective in developmental models of schizophrenia [100]. In addition, these findings underscore the important role of mTOR dysregulation as a molecular mechanism underlying THC-induced neurodevelopmental pathophysiology [54,81].

Many studies have also investigated the potential protective properties of CBD, due to its antipsychotic profile and apparent lack of deleterious side-effects. Indeed, CBD has been found to induce opposite effects on various neurophysiological measures when compared with THC [101], resulting in a promising therapeutic treatment for various neuropsychiatric disorders. For example, previous evidence has demonstrated that intra-NASh CBD administration ameliorated amphetamine-induced deficits in PPI, hyperlocomotion, and increased VTA DA activity [102]. A potential mechanism underlying these CBD effects has been suggested by Norris and colleagues [103], which have reported that CBD decreases spontaneous DAergic firing and bursting rates via functional interaction with the 5HT_1A_ receptor system. In addition, adolescent CBD in spontaneous hypertensive rats prevented the emergence of hyper-motility as well as impairments in sensorimotor gating and contextual fear memory, through modulation of the serotonin system [104]. Notably, therapeutic properties of CBD have been reported in chronic cannabis users, in which prolonged treatment significantly improved memory and cognition as well as decreased depressive-like symptoms, without inducing any side effects [105]. Moreover, CBD was found to normalize the anatomical and neurochemical hippocampal aberrations associated with chronic cannabis consumption [106,107]. Similar preventive effects of CBD on long-term schizophrenia-like symptoms have been also reported in pre-clinical investigations. For example, Murphy and colleagues found that CBD and THC co-administration in adolescent mice prevented deficits in working memory, anxiety, and compulsive-like behaviors [108] (Table 1). Although the mechanisms are not yet fully elucidated, CBD may either antagonize THC effects via negatively modulating an allosteric site of CBRs [109] or interacting with other molecular substrates [101,110,111,112]. For instance, CBD has been found to exert its therapeutic properties by increasing serum levels of endogenous AEA, potentially through inhibition of the enzyme fatty acid amide hydrolase (FAAH) [113]. In support of this finding, administration of the FAAH inhibitor URB597 in adult female rats was able to rescue cognitive and depressive-like symptoms induced by adolescent THC exposure. Such effects, mediated by CB1R signaling, were associated with a higher density of cortical CB1Rs and restoration of THC-related functional aberrations occurring in PFC and DG [70,87] (Table 1). Moreover, increased levels of AEA may activate nuclear peroxisome proliferator-activated receptors alpha (PPARα), an isoform of the nuclear ligand-activated transcription factors family, which has been previously demonstrated to provide neuroprotective effects when measured in several neurodevelopmental models [114,115]. 

As previously mentioned, several genetic polymorphisms may be associated with increased susceptibility for cannabis-related neurodevelopmental pathology. For example, transgenic mice with dominant-negative disrupted in schizophrenia 1 (DN-DISC1) mutations exposed to THC during late adolescence, exhibited anxiolytic behaviors and memory impairments at adulthood, correlated with reduced synaptic plasticity in HC. Notably, wild-type (WT) but not DN-DISC1 THC-treated mice, showed a robust increase of BDNF levels, suggesting that the BDNF response may be a protective mechanism against environmental insults, which fail when other vulnerability factors co-exist. Indeed, BDNF over-expression selectively in DN-DISC1-THC mice was found to prevent the cognitive deficits [116] (Table 1). In addition, a more recent study suggested that such THC-induced memory impairments are mediated by astrocytic mechanisms. For example, adolescent THC treatment in mice expressing DN-DISC1 selectively in astrocytes, activates the proinflammatory pathway NF-kB-COX-2, leading to increased glutamate levels and decreased parvalbumin-positive boutons in HC. Remarkably, selective inhibition of COX-2 signaling, aimed to counteract this neuroinflammatory state, prevented the emergence of these cognitive and glutamatergic abnormalities following adolescent THC exposure [117] (Table 1). 

Further investigations have suggested potential protective properties though the modulation of Neuregulin 1 (Nrg1), another gene related to schizophrenia and cannabis-induced neuropsychiatric effects. For example, Long and colleagues [118] reported that a transgenic model of mice heterozygous in the Nrg1 transmembrane domain (Nrg1 HET) exhibited less susceptibility to anxiety following adolescent acute THC exposure, while chronic THC did not reduce exploratory sniffing behavior during social interaction, as observed in WT control groups. Extending these behavioral effects, THC induced different consequences in the binding density of CB1 and 5-HT_2A_ receptors between Nrg1 HET and WT groups in brain areas related to schizophrenia. In addition, Nrg1 HET THC-exposed mice showed differential expression of hippocampal proteins associated with NMDA receptor trafficking, excessive glutamatergic transmission, excitotoxity and apoptosis when compared with WT mice [119] (Table 1). These observations suggested that the Nrg1 mutation may exert some protective effects toward THC-induced neurodevelopmental pathology.

Lastly, interventions targeting the epigenetic mechanisms involved in THC-related neurodevelopmental abnormalities have been proposed as a potential therapeutic target. In particular, THC exposure in adolescent female rats was found to significantly enhance a marker of the cortical repressive histone H3, through upregulation of the histone-modifying enzyme Suv39H. Co-administration during adolescence of a selective blocker of this enzyme was able to prevent the THC-induced cognitive impairments by normalization of these selective histone modifications [69] (Table 1). Notably, H3 methylation plays a crucial role in normal brain development and GABAergic neurotransmission [120], which raise the possibility that modulation of the histone H3 may restore the cortical inhibitory/excitatory imbalance induced by adolescent THC exposure. Future studies are required to further clarify these possibilities.

## 6. Conclusions

As presented in this review, a large and growing body of evidence has demonstrated that chronic cannabis exposure during adolescence is associated with an increased vulnerability for various neuropsychiatric disorders and cognitive abnormalities. Such disturbances can include impairments in memory and learning as well as schizophrenia-related psychosis, depressive phenotypes, and anxiety disorders. Investigations into the neurodevelopmental exposure to THC in translational animal models have been remarkably consistent with clinical findings and have provided an insight into various neural pathways and biomarkers involved in THC-related pathological outcomes, pinpointing several molecular targets for novel pharmacotherapeutic approaches. In particular, recent findings from our laboratory demonstrated that normalization of the hyper-DAergic state and restoration of the excitatory/inhibitory balance in the mesocorticolimbic system have been successful strategies for preventing and/or reversing the long-lasting behavioral, neural, and molecular consequences induced by adolescent THC [59,96]. 

Given the exponential increase of cannabis use among youth, it is becoming increasingly important to develop safer THC formulations for minimizing potential side effects as well as implementing tiered early interventions. The long-term outcomes related to cannabis exposure during adolescence are complex and can be the result of multiple factors. Specifically, not only THC exposure itself during brain development increases vulnerability for psychiatric disorders, but co-occurrence with other multivariate factors may heighten these risks (see [121] for an extensive review). For instance, while some genetic polymorphisms have been reported in this review as protective factors, others have been found to induce adverse and synergistic effects and increase the vulnerability to cannabis-related developmental insults. In addition, the emerging findings on early-life interferences, such as maternal deprivation or immune system activation, and adolescent THC exposure [122,123], highlight the complexity of such interactions and the need for further investigations.

This complementary evidence underscores the importance of continuing to identify the mechanisms associated with comorbid cannabis use and mental health disorders, in order to develop targeted therapeutic strategies. Moreover, preventive measures and effective health policy will continue to be crucial adjuncts in order to improve public awareness concerning potential neurodevelopmental consequences of cannabis exposure.

## Figures and Tables

**Table 1:** Summary of pre-clinical findings on therapeutic approaches for abnormalities induced by chronic THC during adolescence.

Therapeutic Intervention	THC Adolescent Treatment	Outcomes ^1^	
Muscimol 500 ng/0.5 μL intra-PFC infusions (male SD rats, >PND 75)	THC 2.5–10 mg/kg i.p. twice daily (male SD rats, PND 35–45)	↑ novel object recognition index ↑ social motivation and recognition scores ↑ locomotor activity ↓ anxiety ↓ VTA DA firing activity ↓ VTA DA bursting activity	[59]
L-theanine 10 mg/kg i.p. twice daily (male SD rats, PND 35–45)	THC 2.5–10 mg/kg i.p. twice daily (male SD rats, PND 35–45)	↑ sensory motor gating ↑ social memory ↑ novel object recognition index ↑ sucrose preference ↓ anxiety ↓ VTA DA firing activity ↓ PFC pyramidal bursting rate ↑ PFC gamma oscillations ↑ p-GSK3α-β ↑ p-GSK3α-β/t-GSK3α/β	[96]
SB258585 2.5 mg/kg or rapamycin 1.5 mg/kg i.p. daily (male C57BL/6J mice, PND 30–45)	THC 5 mg/kg i.p. daily (male C57BL/6J mice, PND 30–45)	↓ PFC mTOR ↓ PFC p-p70s6K ↑ object discrimination index ↑ sociability and social discrimination index ↑ PFC mIPSC frequency ↓ PFC mEPSC frequency ↓ PFC resting membrane potential ↑ action potential threshold ↑ rheobase ↓ LTD	[99]
CBD 3 mg/kg i.p. daily (male CD1 mice, PND 28–48)	THC 3 mg/kg i.p. daily (male CD1 mice, PND 28–48)	↑ object discrimination index ↓ compulsive behaviors ↓ anxiety	[108]
URB597 0.3 mg/kg i.p. daily (female SD rats, >PND 75)	THC 2.5–10 mg/kg i.p. twice daily (female SD rats, PND 35–45)	↓ immobility, ↑ swimming in FST ↑ sucrose consumption ↑ social interaction = recognition memory	[70]
URB597 0.3 mg/kg i.p. daily (female SD rats, >PND 75)	THC 2.5–10 mg/kg i.p. twice daily (female SD rats, PND 35–45)	↑ PFC CB1Rs ↑ PFC LTD ↑ DG dendritic arborization ↑ DG DCX^+^ cells ↓ immobility, ↑ swimming in FST	[87]
BDNF over-expression in dorsal HC	THC 10 mg/kg i.p. daily (male DN-DISC1 C57BL/6J mice, PND 42–51)	↑ object recognition memory = locomotor activity = anxiety	[116]
NS398 10 mg/kg s.c. daily (male and female astrocytic DN-DISC1 mice, PND 30–51) or NS398 20 μM in culture medium	THC 8 mg/kg s.c. daily (male and female astrocytic DN-DISC1 mice, PND 30–51) or THC 5 mM in culture medium	↑ spatial memory ↑ novel object recognition index ↑ novel place recognition index ↑ glutamate secretion	[117]
Genetic modification in Nrg1 gene	THC 10 mg/kg i.p. daily (male Nrg1 HET mice, PND 30–51)	= locomotor activity = anxiety = PPI and startle reflex ↑ sniffing behavior ↑ Substantia nigra CB1Rs binding ↑ Anterior insula 5HT_2A_ binding	[118]
Genetic modification in Nrg1 gene	THC 10 mg/kg i.p. daily (male Nrg1 HET mice, PND 30–51)	↑ APOA_1_ ↓ GPSM_2_ ↓ FLOT_1_	[119]
Chaetocin 0.05 mg/kg i.p daily (female SD rats, PND 35–45)	THC 2.5–10 mg/kg i.p. twice daily (female SD rats, PND 35–45)	↑ novel object recognition index = social behavior = immobility, = swimming in FST	[69]

^1^ Outcomes of therapeutic interventions when compared with THC adolescent treated groups.

## Data Availability

Not applicable.
